# Mutations in fibroblast growth factor (FGF8) and FGF10 identified in patients with conotruncal defects

**DOI:** 10.1186/s12967-020-02445-2

**Published:** 2020-07-14

**Authors:** Shuang Zhou, Qingjie Wang, Zhuo Meng, Jiayu Peng, Yue Zhou, Wenting Song, Jian Wang, Sun Chen, Kun Sun

**Affiliations:** Department of Pediatric Cardiology, Xinhua Hospital, School of Medicine, Shanghai Jiao Tong University, No. 1665, Kongjiang Road, Yangpu District, Shanghai, 200092 China

**Keywords:** Conotruncal defect, Fibroblast growth factor 8, Fibroblast growth factor 10, Target sequencing

## Abstract

**Background:**

Conotruncal defects (CTDs) are a type of heterogeneous congenital heart diseases (CHDs), but little is known about their etiology. Increasing evidence has demonstrated that fibroblast growth factor (FGF) 8 and FGF10 may be involved in the pathogenesis of CTDs.

**Methods:**

The variants of FGF8 and FGF10 in unrelated Chinese Han patients with CHDs (n = 585), and healthy controls (n = 319) were investigated. The expression and function of these patient-identified variants were detected to confirm the potential pathogenicity of the non-synonymous variants. The expression of FGF8 and FGF10 during the differentiation of human embryonic stem cells (hESCs) to cardiomyocytes and in Carnegie stage 13 human embryo was also identified.

**Results:**

Two probable deleterious variants (p.C10Y, p.R184H) of FGF8 and one deletion mutant (p.23_24del) of FGF10 were identified in three patients with CTD. Immunofluorescence suggested that variants did not affect the intracellular localization, whereas ELISA showed that the p.C10Y and p.23_24del variants reduced the amount of secreted FGF8 and FGF10, respectively. Quantitative RT-PCR and western blotting showed that the expression of FGF8 and FGF10 variants was increased compared with wild-type; however, their functions were reduced. And we found that FGF8 and FGF10 were expressed in the outflow tract (OFT) during human embryonic development, and were dynamically expressed during the differentiation of hESCs into cardiomyocytes.

**Conclusion:**

Our results provided evidence that damaging variants of FGF8 and FGF10 were likely contribute to the etiology of CTD. This discovery expanded the spectrum of FGF mutations and underscored the pathogenic correlation between FGF mutations and CTD.

## Background

Conotruncal defects (CTDs) are a complex type of congenital heart diseases (CHDs) with an approximate prevalence of 0.1‰ among live births [1], and approximately 25–30% of all non-syndromic CHDs. CTDs include the following conditions: tetralogy of Fallot (TOF), double outlet of right ventricle (DORV), pulmonary atresia with ventricular septal defect (PA/VSD), transposition of the great arteries (TGA), interrupted aortic arch (IAA), and persistent truncus arteriosus (PTA). CTDs are the most common type of cyanotic CHDs [2]. They usually require catheter-based or surgical treatment early in life, but the mortality rate remains high [3]. The outflow tract (OFT) is a conduit through which blood flows from the ventricles into the pharyngeal arch arteries (PAA) and their derivatives [4]. The OFT plays a vital role in normal cardiac development. During embryogenesis, the OFT undergoes a series of elaborate remodeling processes, including the development of the secondary heart field (SHF) and the cardiac neural crest (CNC) [5], which forms the basis of the aorta and pulmonary artery [4]. Although increasing studies have shown the major role of genetic factors in the pathogenesis of CTDs, the underlying mechanisms of genetic determinants remain unclear [6, 7].

Fibroblast growth factors (FGFs) are a group of secreted signaling proteins composed of 22 members, regulating development, metabolism, and homeostasis [8–10]. FGFs have vital roles in normal cardiac morphogenesis [11]. FGF8 and FGF10 are members of the FGF family, playing a role in paracrine signaling during the development of the embryonic heart [12]. FGF8 has been shown to be the major FGF ligand driving the development of both the SHF and CNC [13, 14]. Moreover, FGF8 secreted by SHF progenitor cells is thought to be involved in the interaction between the SHF and CNC [15]. It has been reported that FGF8 is expressed and plays a role in the splanchnic mesoderm, cardiac crescent, pharyngeal endoderm and ectoderm during the development of OFT and pharyngeal arches. Accordingly, embryos of FGF8 knockout mouse were reported to die early, and even conditional knockouts showed severe OFT and right ventricle (RV) defects [13, 14, 16, 17]. Likewise, FGF10, a specific endogenous marker of the SHF [18], is an important regulator of the proliferation of differentiated cardiomyocytes in developing embryos. Pharyngeal mesoderm expressing FGF10 has been shown to give rise to the arterial pole of the heart [18] and finally contributes to the formation of the OFT and RV of the mammalian heart. However, although at this stage the heart is ectopic and the pulmonary artery and vein are absent, formation of the OFT proceeds normally in mice lacking FGF10 [19]. Nevertheless, the effects on the development of OFT are exacerbated in FGF8 and FGF10 compound mutants [20, 21]. In summary, several studies indicated that FGF8 and FGF10 are essential for proliferation of the SHF and normal morphogenesis of the heart, and have partially overlapping functions during formation of the OFT.

Here, using target sequencing, we reported two nonsynonymous variants of FGF8 (p.C10Y and p.R184H) and one deletion mutant (p.23_24del) of FGF10 in three CTD patients. Our results showed that these variants led to the increased mRNA and protein expression of both FGF8 and FGF10, but their functions were relatively reduced. This discovery broadened the spectrum of FGF mutations and further elucidated the genetic pathogenesis of CTDs.

## Materials and methods

### Study population and DNA isolation

Our study population included 585 unrelated patients with CTD of Han ethnicity, who were diagnosed either by echocardiogram or examination from a cardiologist or surgeon from Shanghai Children’s Medical Center affiliated to Shanghai Jiao Tong University School of Medicine. All subjects were unrelated, with an age ranging from newborn to 16 years old (Table 1). The gene loci of all CTD patients were detected by CNVplex^®^ (a technique for the high throughput detection of sub-chromosomal copy number aberrations) as previously described [22] to exclude patients with known syndromes or chromosomal abnormalities. In addition, 391 unrelated healthy individuals without CHD of Han ethnicity, were included as a control group (data not shown). Peripheral blood samples were collected from all study subjects for DNA extraction. The extraction of genomic DNA was performed using the QIAamp DNA Blood Mini Kit (QIAGEN, Germany) following a standard phenol–chloroform extraction protocol and was then stored at − 80 °C until used.

**Table 1:** Cardiac diagnoses for study patients with CTDs

Diagnosis	Number	Percentage	Age
TOF	224	38.3	1 month–14 years
DORV	99	16.9	25 days–16 years
PA/VSD	95	16.3	3 month–12 years
TGA	85	14.5	0 day–16 years
SA/SV	46	7.9	24 days–12 years
IAA	13	2.2	6 days–1 years
PA + IVS	13	2.2	2 days–3 years
PTA	10	1.7	2 days–2 years
Total	585	100	0 day–16 years

TOF, tetralogy of Fallot; DORV, double outlet of right ventricle; PA/VSD, pulmonary atresia with ventricular septal defect; TGA, transposition of the great arteries; SA, single atrium; SV, single ventricle; IAA, interrupted aortic arch; PTA, persistent truncus arteriosus

### Screening for mutations and variant analysis

Target sequencing refers to the enrichment of target region by hybridization or augmentation, through next generation sequencing method combined with biological information in the target area for assembly and sequence analysis. Our target sequencing only detected genes exons related to cardiac development. Target sequencing of FGF8 (GenBank accession number NC_000010.11, NM_033163) and FGF10 (GenBank accession number NC_000005.10, NM_004465) mutants was performed using the Illumina HiSeq 2000 platform. Candidate variants were then confirmed by Sanger sequencing. Primers for the PCR amplification of FGF8 and FGF10 were designed using Primer Premier 5 (Additional file 1: Table S1). Using the GenBank BLAST program (http://blast.ncbi.nlm.nih.gov/Blast.cgi) to compare the sequence traces with the FGF8 and FGF10 reference sequence. We ruled out the influence of other possible genes. The verified sequence variants were all queried in the Exome Aggregation Consortium database (ExAC, http://exac.broadinstitute.org), 1000 Genomes database (http://www.1000genomes.org), SNP database at the National Center for Biotechnology Information (NCBI; http://www.ncbi.nlm.nih.gov) and Ensembl database (http://asia.ensembl.

org). We used several bioinformatics websites, including Polyphen-2 (http://genetics.bwh.havard.edu/pph2/), SIFT (http://sift.jcvi.org/www/SIFT_enst_submit.html), and Mutation Taster (http://www.mutationtaster.org/), to predict the effect of nonsynonymous variations (Table 2).

**Table 2:** Characteristics of missense variants identified in *FGF8* and *FGF10*

Patient	Age	Diagnosis	Gene	Location in gene	Function	Amino acid change	SIFT	Mutation taster	PolyPhen-2
F150	6 months	TOF	*FGF8*	Exon1	Benign	29G>A	0.88	Polymorphism	0
F059	1 year	TOF/PFO/ASD	*FG8*	Exon6	Probably damage	551G>A	0	Disease causing	0.999
S033	5 months	SA/SV/CAVC/PS/PH	*FGF10*	Exon1	–	68_70del	–	Disease causing	–

TOF, tetralogy of Fallot; PFO, patent foramen ovale; ASD, atrial septal defect; SA, single atrium; SV, single ventricle; CAVC, complete atrioventricular canal; PS, pulmonary stenosis; PH, pulmonary hypertension

### Homology analysis of FGF8 and FGF10 protein sequences

ClustalX software was used to analyze the homology of FGF8 and FGF10 protein sequences. The FGF8 and FGF10 protein sequences of Homo sapiens (human), Mus musculus, Pan troglodytes, Bos Taurus, Equus caballus, Macaca mulatta, Ovis aries, and Sus scrofa were downloaded from NCBI (https://www.ncbi.nlm.nih.gov/protein/).

### Construction of plasmids and design of mutants

The human FGF8 (pCMV6-Entry-FGF8, Myc-DDK-tagged) and FGF10 (pCMV6-Entry-FGF10, Myc-DDK-tagged) cDNA plasmids were purchased from Origene (Rockville, MD, USA). Mutated primers were designed to amplify human FGF8 and FGF10 cDNA according to the protocol provided by the QuickChange II Site-Directed Mutagenesis kit (Stratagene, USA), and then, the cDNA of FGF8 and FGF10 variants was amplified by polymerase chain reaction (PCR) and introduced into wild type pCMV6-Entry vectors at the SgfI and MluI restriction sites. The sequences of wild-type and variant inserts were confirmed by performing Sanger sequencing, ensuring the lack of further base exchanges in the variant sequences.

### Transient transfection

Human cardiomyocytes (HCM) and HEK 293T cells (human embryonic kidney cells) were cultured in Dulbecco Modified Eagle’s Medium (DMEM, Invitrogen, CA, USA) supplemented with 10% fetal calf serum (FBS, MP Biomedicals, USA), as well as 100 U/mL of penicillin and 100 μg/mL of streptomycin (Gibco, USA) at 37 °C in an atmosphere of 5% CO_2_ and 95% air. All transient transfections in HCM and HEK 293T cells were performed using the FuGene HD (Promega, USA) according to the manufacturer’s protocol.

### Western blot analysis

Plasmids were transfected into HCM and HEK 293T cells seeded in 12-well plates. Cells were harvested 48 h after transfection, and lysed in RIPA lysis buffer (Beyotime, China) supplemented with PMSF (1:100). After boiling for 10 min, cell lysates were analyzed by sodium dodecyl sulfate polyacrylamide (SDS-PAGE) gel electrophoresis and transferred onto nitrocellulose blotting membranes (GE Healthcare). Membranes were blocked with 5% skim milk for 2 h at 25 °C and then incubated with anti-human FGF8 (1:2000; p102085, KleanAB), anti-human ETV4 (1:2000, AB33049), anti-human FGF10 (1:2000; AB32224, absci), or anti-human FGFR2 (1:2000) antibodies, as well as with anti-GAPDH (1:5000, ab8245, Abcam) or anti-actin antibodies (1:10,000; ab3280, Abcam) overnight at 4 °C. Membranes were then incubated with horseradish peroxidase-conjugated anti-rabbit (1:3000) and anti-mouse (1:3000) secondary antibodies. Protein bands were visualized using a chemiluminescent HRP substrate (Millipore, MA, USA) and analyzed using the Image Lab software (BioRad, Philadelphia, PA, USA).

### Quantitative RT-PCR

HCM and HEK 293T cells seeded in 12-well plates were transfected with a total of 1.1 µg of wild-type or variant plasmid DNA. Cells were harvested 36 h after transfection. TRIzol reagent (Invitrogen, USA) was used to extract total RNA. Subsequently, the extracted RNA was reverse transcribed to cDNA using the Prime Script RT Master Mix (Takara, Japan), followed by quantitative RT-PCR analysis using the TB Premix Ex Taq (Takara) on an Applied Biosystems 7500 system (Applied Biosystems, USA). The 2^−ΔΔCt^ method was used to calculate the relative expression of genes [23], using the human glyceraldehyde-3-phosphate dehydrogenase gene (*GAPDH*) as an internal control. Primer sequences are listed in Additional file 1: Table S2.

### Immunofluorescence assay

HCM were seeded onto a 12-well plate covered with poly-l-lysine (0.1 mg/mL). After 24 h of incubation, cells were transfected with wild-type or variant plasmids. Cells were harvested 24 h after transfection, permeabilized for 10 min using 0.3% Triton X-100/PBS, and blocked with 5% BSA/PBS for 1 h at 25 °C. Consecutively, cells were incubated with either a rabbit anti-human FGF8 (1:100; KleanAB) antibody, or a rabbit anti-human FGF10 (1:100; AbSci) antibody diluted in 1% BSA/PBS at 4 °C overnight, followed by incubation with Cy3-conjugated goat anti-rabbit (1:200) secondary antibody for 2 h at 37 °C. Nuclei were then stained with 4, 6-diamidino-2-phenylindole (DAPI; Vector Laboratories, USA) for 7 min at 25 °C. An Olympus BX43 microscope (Olympus, Shinjuku-ku, Tokyo, Japan) was used for image acquisition and analysis.

### Cell viability assay

A Cell Counting Kit-8 (CCK-8; MedChem Express, Monmouth Junction, NJ, USA) was used to assess the effect of the wild-type or variant FGF8 and FGF10 constructs on the viability of cells, following the manufacturer’s protocol. HCM cells were seeded in 96-well plates (2000 cells/well). After 24 h, cells were transfected with a total of 110 ng of either pCMV6-Entry, wild-type, or variant plasmids. After an additional 40–48 h incubation, the cell medium was replaced with FBS-free medium containing 10% CCK-8 solution and incubated for 1.5 h at 37 °C. Absorbance values were measured at 450 nm using a microplate reader (BioTek, USA). Cell viability was calculated as follows: (OD_450_samples − OD_450_blank)/(OD_450_control − OD_450_blank) ×  100%.

### Enzyme-linked immunosorbent assays (ELISA)

Double-antibody sandwich enzyme-linked immunosorbent assays (ELISA) were used to evaluate the secretory capacity of HCM transfected with the variants compared with wild-type constructs. After 48 h of transfection, the culture media were collected and centrifuged for 10 min at 3000 rpm to remove cells and polymers according to the manufacturer’s instructions (YAD, China). Consecutively, 10 μL of each supernatant was transferred to an ELISA microtiter plate supplemented with 40 μL of sample dilution buffer, and 50 μL of HRP-conjugated antibody. Reaction wells were sealed with a sealing membrane and incubated for 60 min at 37 °C in an incubator. Following incubation, the liquid was discarded, the plate was dried on absorbent paper and each well was washed for 1 min five times. Then, 50 μL of each of substrate A and B were added to each well, and incubated at 37 °C for 15 min in the dark. Finally, 50 μL of stop solution was added to all wells and the optical density value of each well was immediately measured at a wavelength of 450 nm using a standard microplate reader (BioTek, USA). The amount of FGF8 protein in the supernatants was calculated using a standard curve generated from standards with known concentrations.

### Differentiation of human embryonic stem cells

To simulate cardiac development in vitro, we induced directed differentiation of human embryonic stem cells (hESCs) into cardiomyocytes. hESCs were initially cultured in mTeSR1 medium until the fusion rate was approximately 90%. After that, the culture medium was replaced with RPMI 1640 (Corning). On day 0 to 1, 12 mM of CHIRi-99021 (Selleck) was added to the medium. After 24 h, IWR-1 (5 mM; Sigma) was added to fresh RPMI medium. On day 5 to 6, the medium was changed to RPMI/B-27 containing insulin. Then, fresh RPMI/B-27 medium was added every day until the seventh day after differentiation. Cells were kept in an incubator at 37 °C with 5% CO_2_.

### Immunohistochemistry

Human embryos of Carnegie stage (CS) 13 were obtained after termination of pregnancy at the Shanghai Xin Hua Hospital. Embryos were fixed for 16–24 h in 4% paraformaldehyde/PBS solution, embedded in paraffin, and then sectioned at a thickness of 5 μm. For immunolocalization of FGF8 and FGF10, paraffin sections were incubated with a primary rabbit anti-human FGF8 (1:50; KleanAB) and a rabbit anti-human FGF10 antibody (1:50; AbSci) and then incubated with a secondary anti-rabbit antibody (1:200; Abcam) and DAB (Abcam).

### Tissue collection and microarray experiment

Human embryo heart samples at CS10-16 were obtained after pregnancy termination at the Shanghai Xinhua Hospital. The total RNA was extracted using the TissueLyser II (Qiagen) and RNeasy MinElute Cleanup Kit (Qiagen). Then, we performed transcriptome array analysis [24] to detect gene expression levels at different developmental stages. Raw data was normalized by Affymetrix Transcriptome Analysis Console (TAC) software, and the normalized signal value was the signal value calculated by log2 transformation.

### Statistical analysis

Data was presented as mean ± standard deviation (SD). The independent-samples *t* test was used to evaluate the statistical significance of the observed differences between unpaired samples. Statistical differences in the allele frequency between patients with CTD and controls were evaluated using the Chi square test. A difference was considered statistically significant at *p* < 0.05.

## Results

### Variants of *FGF8* and *FGF10* in patients with nonsyndromic CTD

**Fig. 1:**
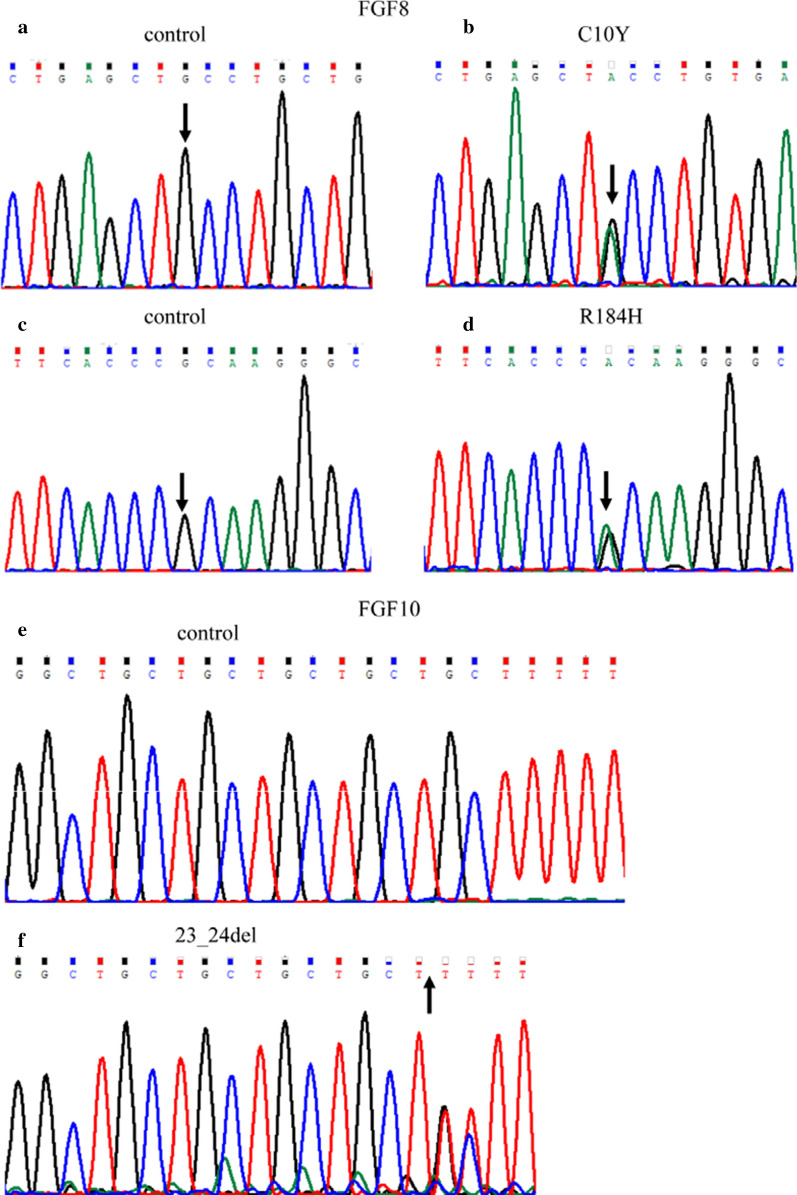
Sequences of FGF8 and FGF10 mutants identified in patients with CTD and controls. **a**, **c**, **e** Chromatograms of normal controls. **b**, **d** Chromatograms of the two heterozygous variants. **f** Deletion mutation in FGF10. Arrows indicate the nucleotide changes and the deletion

Target sequencing only detected genes exons related to cardiac development, we excluded the effects of other possible genes, and finally identified two mutant variants of FGF8 (Fig. 1b, d) in two patients with TOF (Additional file 1: Figure S1a, b) and one deleted variant of FGF10 (Fig. 1f) in a patient with single atrium, single ventricle, complete atrioventricular valve defect, and pulmonary valve stenosis (Additional file 1: Figure S1c). No synonymous mutations were found in these three patients. The allelic frequencies of the *FGF8* p.R184H (NM_033163.4: c.551G>A) and *FGF10* p.23_24del (NM_004465.1: c.68_70del) variants found in the ExAC database were 8.379e-06 and 0.0004322, respectively. Interestingly, the *FGF8* p.R184H variant had been previously found only in the European populations. Notably, the *FGF8* p.C10Y variant had never been previously reported. The characteristics of these variants of the FGF8 and FGF10 proteins are shown in Table 2.

### Comparison of multiple FGF8 and FGF10 protein sequences

**Fig. 2:**
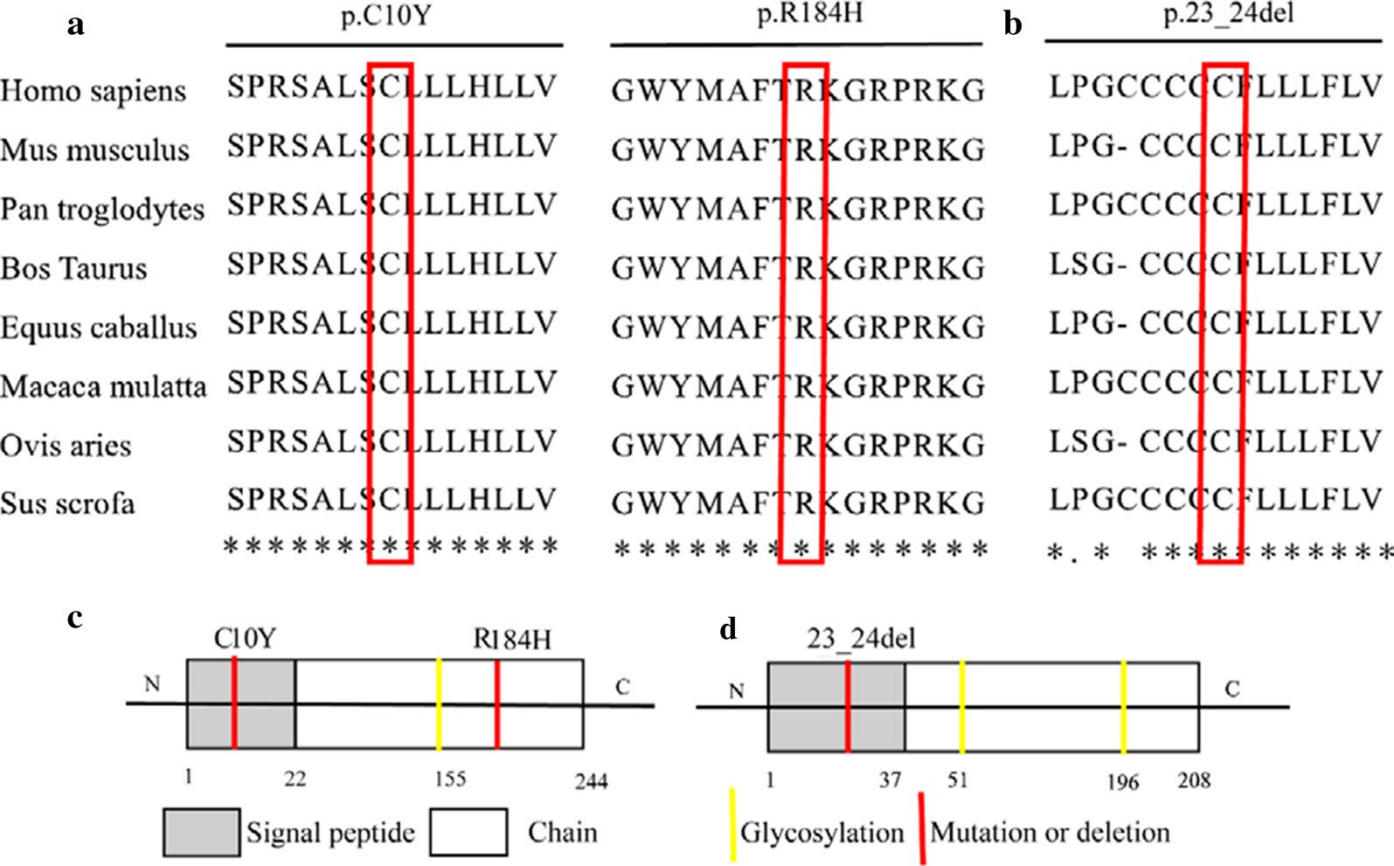
Distribution and conservation of mutations in FGF8 and FGF10. **a**, **b** Alignment of multiple FGF8 and FGF10 protein sequences among species. **c**, **d** Structure of the FGF8 and FGF10 proteins and the location of the genetic variations in this study

The human *FGF8* gene is located on the chromosomal region 10q24 and consists of six exons and five introns, whereas the human *FGF10* gene is located on the chromosomal region 5p12 and consists of four exons and three introns. Alignment of multiple FGF8 and FGF10 protein sequences indicates that these mutation sites are highly evolutionary conserved in mammals (Fig. 2a, b), indicating that these mutations may severely impact the proteins’ functions. Structurally, FGF8 and FGF10 are both paracrine proteins, composed of a secretory signal peptide in the amino-terminal (N-terminal) domain and a mature peptide in the carboxyl terminal (C-terminal). The positions of these variants in the FGF8 and FGF10 proteins are shown in Fig. 2c and d, respectively. The p.C10Y and p.23_24del variations were located in the signal peptide region of FGF8 and FGF10, respectively, whereas the p.R184H mutation was located in the mature peptide of FGF8.

### Expression levels of FGF8 and FGF10 mutations

To evaluate the effect of the identified mutations on the expression level, we overexpressed the wild-type and mutated FGF8 and FGF10 in HCM and HEK 293T cells, and then performed western blotting and quantitative RT-PCR analysis according to the manufacturer’s protocol. Results showed that the mutated FGF8 and FGF10 were expressed at higher levels than the wild-type version at both the mRNA (Figs. 3a, c, 4a, c) and the protein (Figs. 3b, d, 4b, d) levels.

**Fig. 3:**
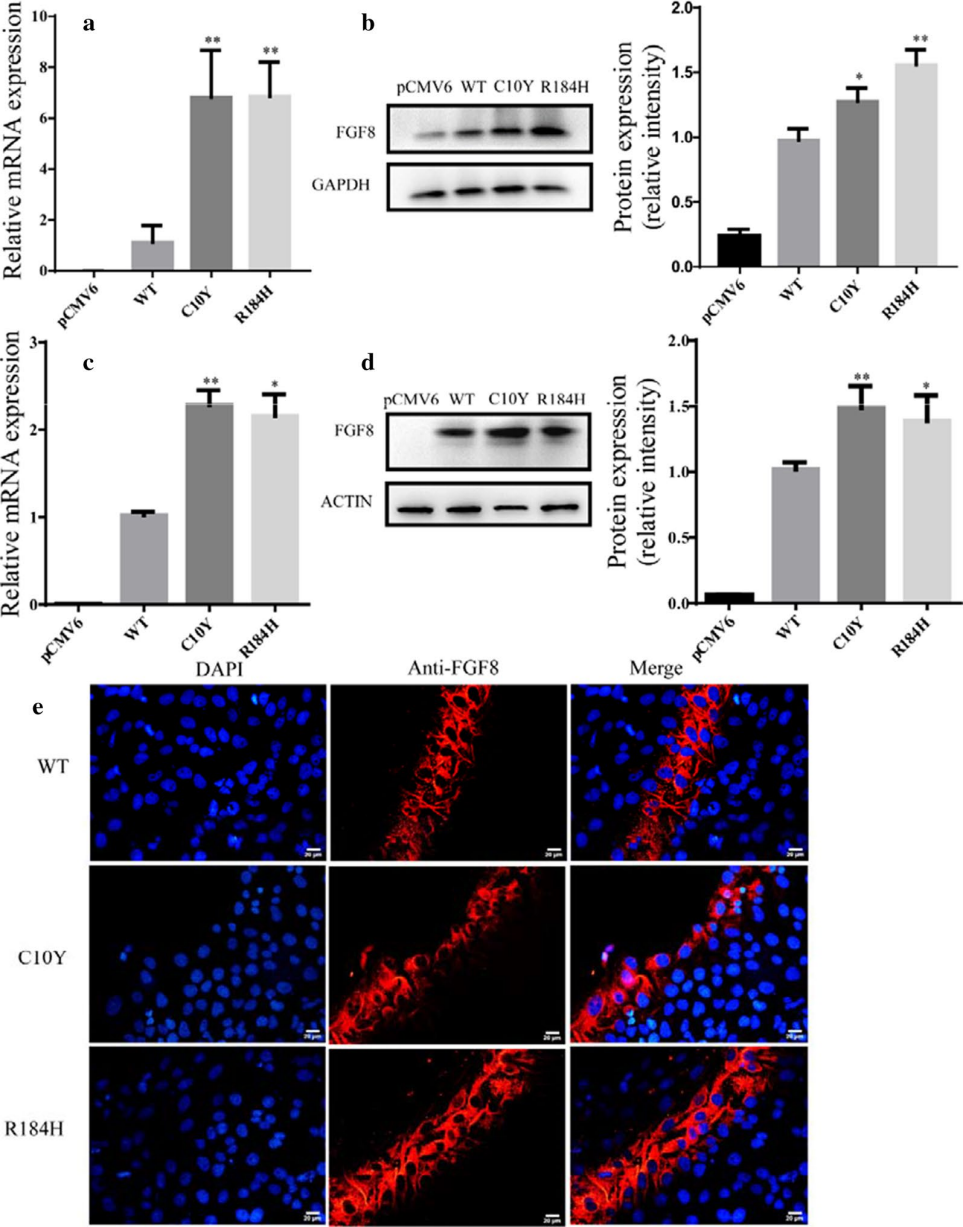
The expression and intracellular localization of wild-type and mutated FGF8. Relative mRNA expression of wild-type and variants of FGF8 in HCM (**a**) and HEK 293T (**c**), respectively (n = 3). GAPDH was used as an internal control. Western blot analysis and density quantitation in HCM (**b**) and HEK 293T (**d**) transfected with the blank vector, wild-type, and mutated FGF8. GAPDH and β-actin were used as an internal control in HCM and HEK293T, respectively (n = 3). **e** Immunofluorescence staining of wild-type and variants of FGF8. Images represented here were obtained from 3 biological replicates. Scale bar 20 μm

**Fig. 3:**
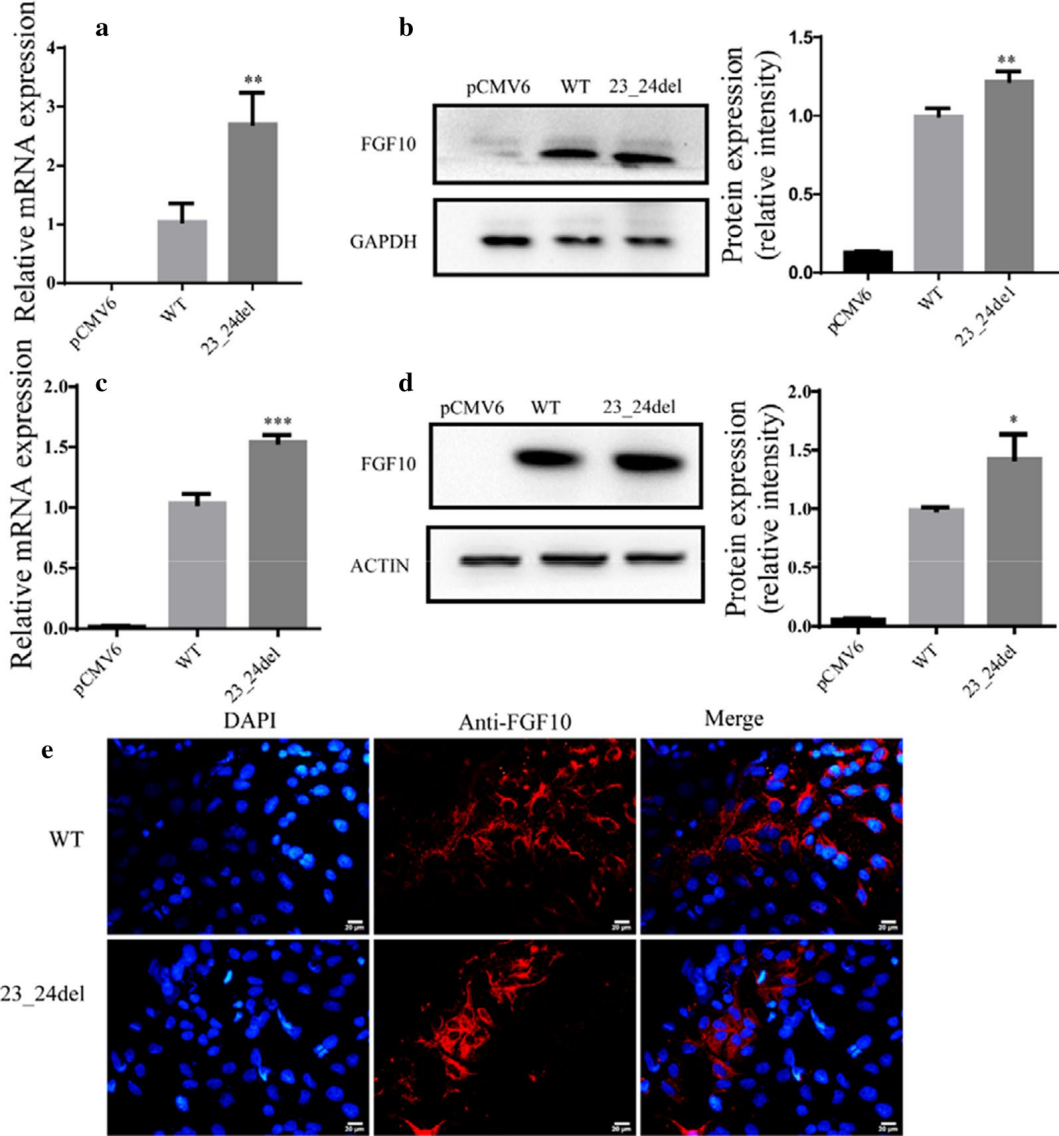
The expression and intracellular localization of wild-type and mutated FGF10. Relative mRNA expression of wild-type and variants of FGF10 in HCM (**a**) and HEK 293T (**c**), respectively (n = 3). GAPDH was used as an internal control. Western blot analysis and density quantitation in HCM (**b**) and HEK 293T (**d**) transfected with the blank vector, wild-type, and mutated FGF10. GAPDH and β-actin were used as an internal control in HCM and HEK293T, respectively (n = 3). **e** Immunofluorescence staining of wild-type and variants of FGF10. Images represented here were obtained from 3 biological replicates. Scale bar 20 μm

### Intracellular localization of FGF8 and FGF10 mutations

The intracellular localization of FGF8 and FGF10 mutated proteins was analyzed by immunofluorescence assays. Our results showed that none of these variants affected the intracellular localization, and both the wild-type and mutated proteins were expressed in the cytoplasm (Figs. 3e, 4e). Thus, we hypothesized that the mutations might change the protein function through other mechanisms.

### Functional analysis of FGF8 and FGF10 variant proteins

**Fig. 5:**
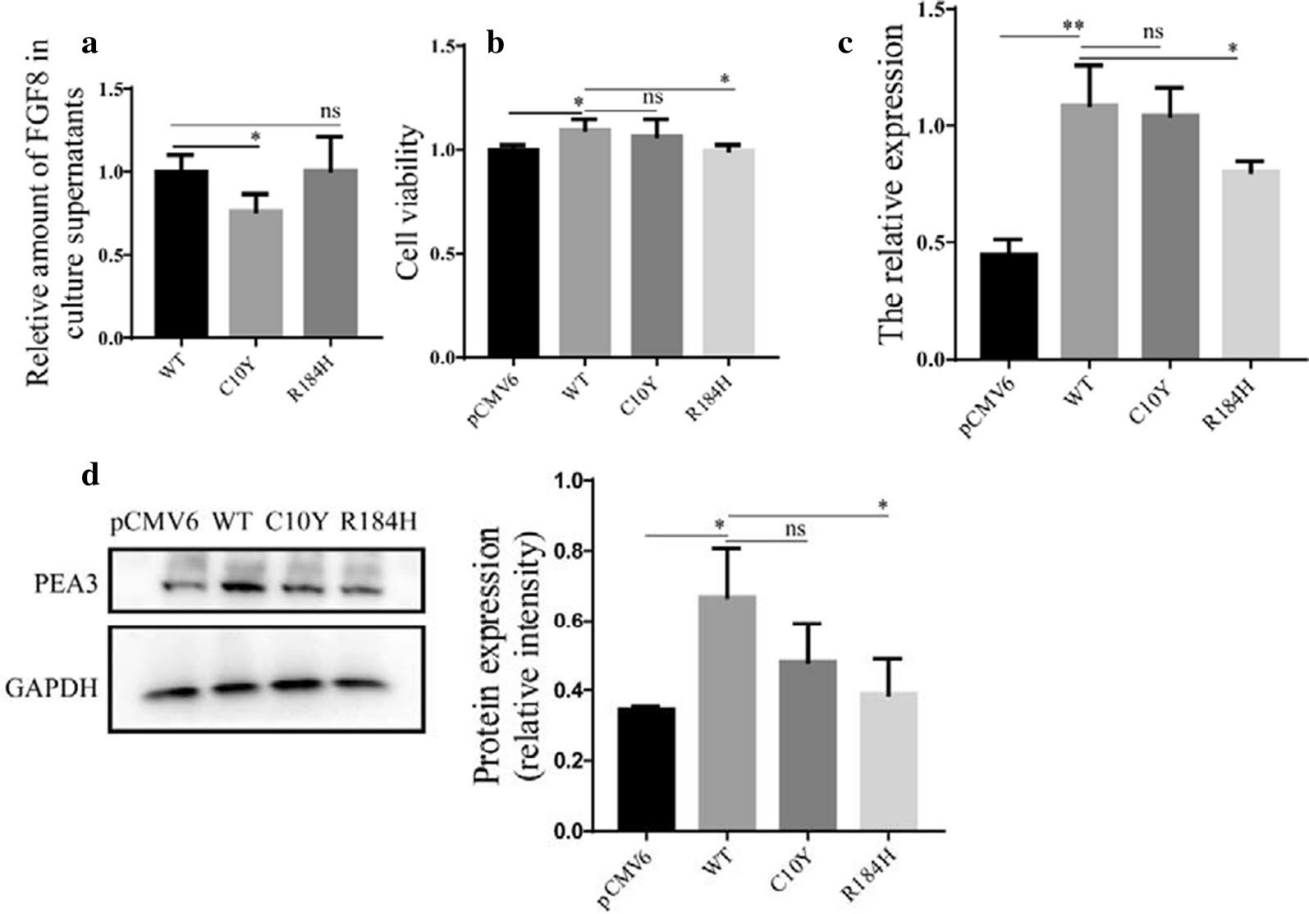
Functional analysis of wild-type and mutated FGF8. **a** Relative amount of FGF8 in supernatants. **b** Effects of mutant and wild-type FGF8 on cell viability. **c**, **d** The variant effect of FGF8 on PEA3 expression (n = 3), GAPDH was used as an internal control

**Fig. 6:**
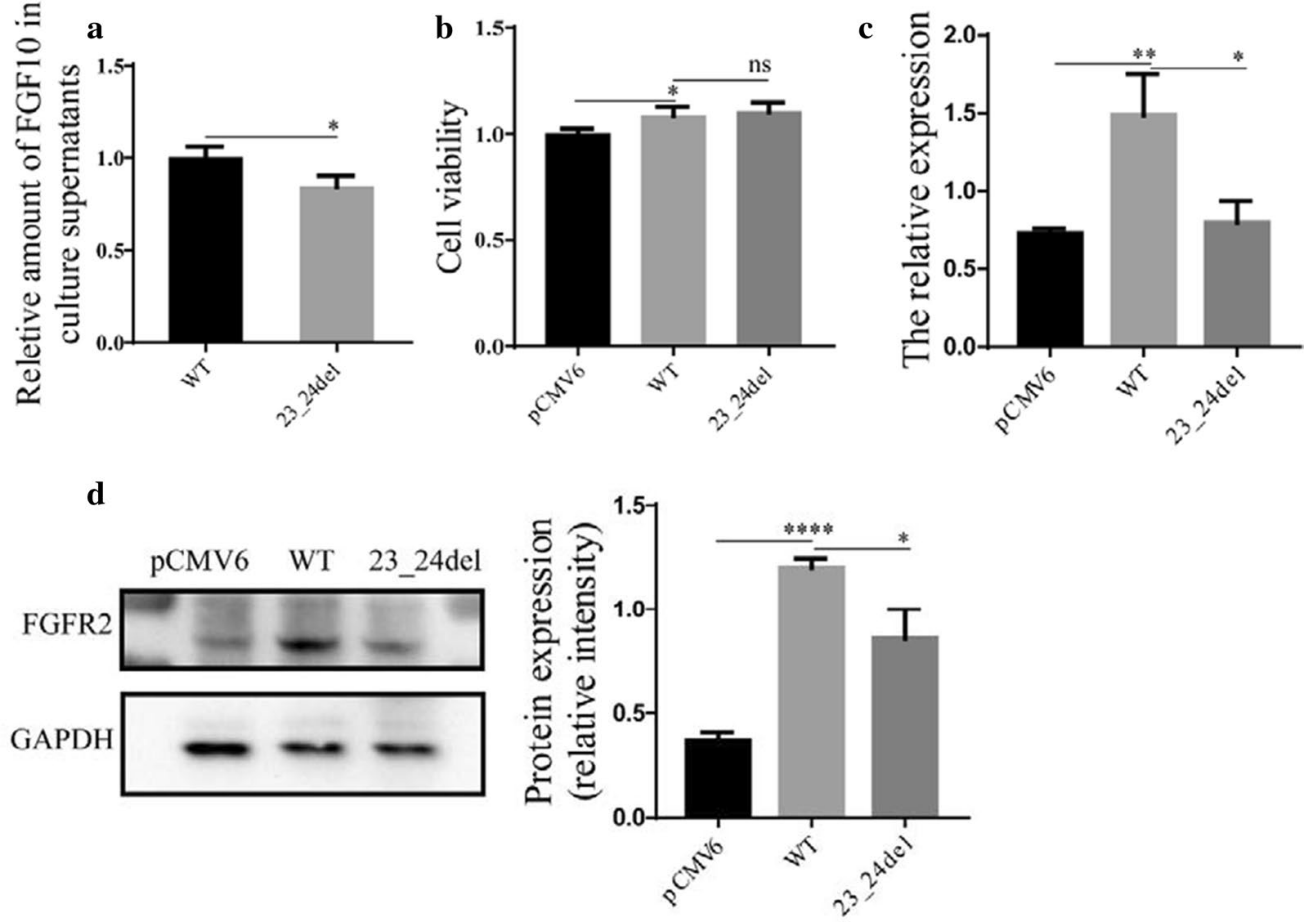
Functional analysis of wild-type and mutated FGF10. **a** Relative amount of FGF10 in supernatants. **b** Effects of mutant and wild-type FGF10 on cell viability. **c**, **d** The variant effect of FGF10 on FGFR2 expression (n = 3), GAPDH was used as an internal control

To investigate the effect of the mutations on their secretion, we performed ELISA on the culture medium of cells expressing either the wild-type or the mutated FGF8 and FGF10. Our results showed that p.C10Y and p.23_24del mutations decreased the secretion capacity of FGF8 and FGF10, respectively, which might be because they were located in signal peptide regions [25]. On the other hand, the p.R184H mutation did not significantly change the secretion capacity of FGF8 (Figs. 5a, 6a). Moreover, we found that overexpression of FGF8 or FGF10 promoted the proliferation of HCM, whereas mutations minimized their effect on cell proliferation (Figs. 5b, 6b). In addition, we selected several important genes associated with FGF8 or FGF10 during OFT development from previous studies (Additional file 1: Figure S2). We found that overexpression of wild-type FGF8 and FGF10 promoted the expression of ETS variant transcription factor 4 (ETV4, known also as PEA3) (Fig. 5c, d) and fibroblast growth factor 2 (FGFR2) (Fig. 6c, d), respectively, whereas the promotion effect was reduced when the mutated versions were overexpressed. This result suggested that these might be the downstream genes in the pathway regulated by FGF8 and FGF10 and might be correlated to pathogenesis of CTD. Collectively, our results indicated that although the variants increased the intracellular expression of these protein, their functional activity was reduced compared to the wild-type.

### Expression of *FGF8* and *FGF10* during the differentiation of hESCs into cardiomyocytes

**Fig. 7:**
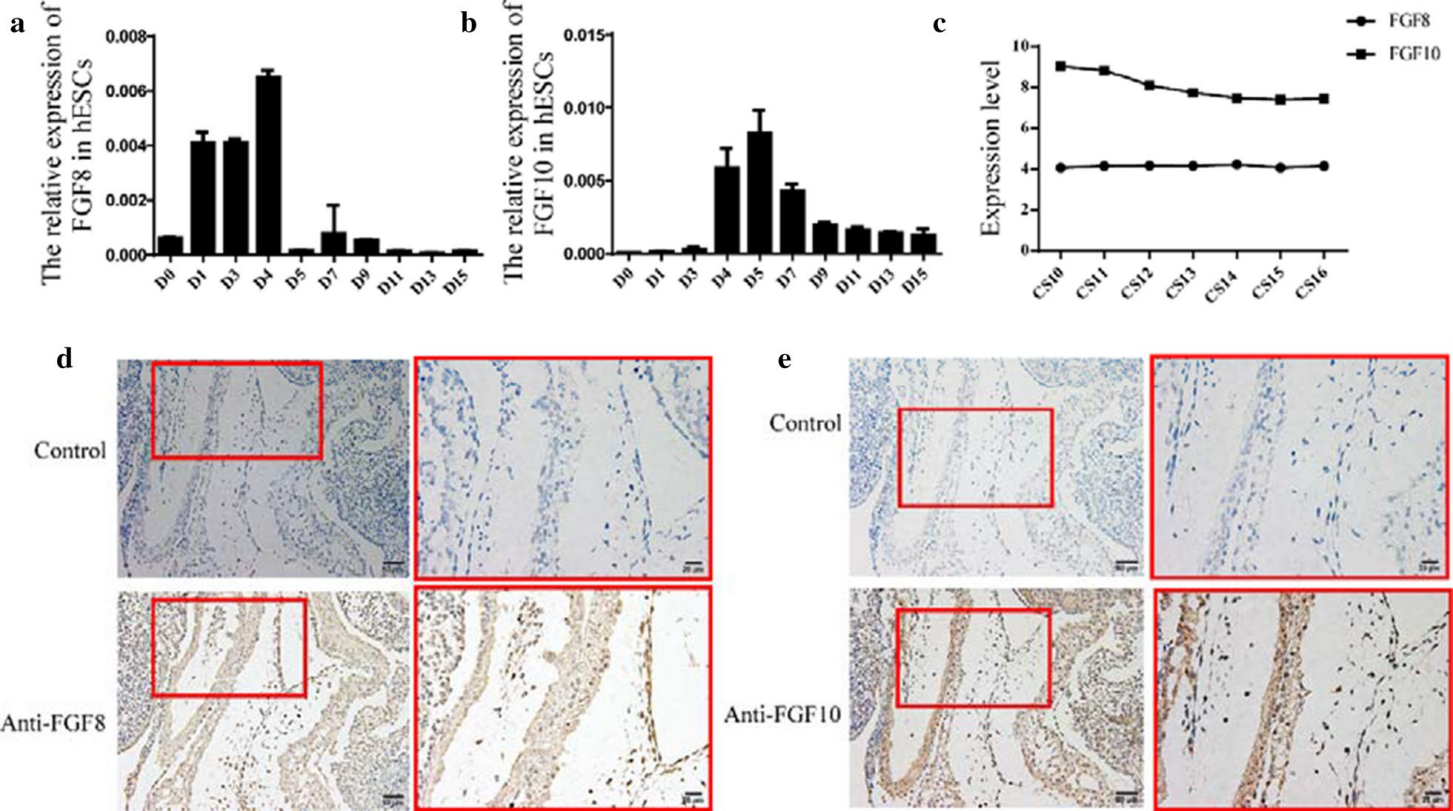
Dynamic expression of FGF8 and FGF10 in human embryonic stem cells and human embryonic cardiac tissue. **a**, **b** Relative expression of FGF8 and FGF10 during the differentiation of human embryonic stem cells to cardiomyocytes. **c** Expression level of FGF8 and FGF10 in the human embryonic heart, **d**, **e** Immunohistochemistry of FGF8 and FGF10 in human embryos at Carnegie stge13

Although FGF8 and FGF10 played important roles in embryonic heart development, their expression had never been identified during the differentiation of hESCs into cardiomyocytes; therefore, we analyzed their expression in these cells by qRT-PCR. We found that the expression of *FGF8* was high in the earlier stages of differentiation, but decreased significantly by the fifth day of the differentiation of hESCs to cardiomyocytes (Fig. 7a). Whereas, the expression of *FGF10* was low in the stem phase of hESCs, increased on the fourth day of the differentiation of hESCs to cardiomyocytes, peaked on the fifth day, and then decreased again (Fig. 7b). The expression patterns of *FGF8* and *FGF10* during the differentiation of hESCs to cardiomyocytes suggested that the functions of these proteins might be different and complementary during the development of the heart.

### Expression level of *FGF8* and *FGF10* in the human embryonic heart

The expression of FGF8 and FGF10 has not been identified in the human embryo. Therefore, we collected human embryonic hearts from CS10 to CS16 and performed gene expression analysis using the human transcriptome array 2.0. The expression levels of FGF8 and FGF10 were represented by the mean of the sample expression levels. Our analysis revealed that both FGF8 and FGF10 were expressed throughout these development stages, and the expression of FGF10 in the heart was higher than that of FGF8 (Fig. 7c). We then performed immunohistochemistry for FGF8 and FGF10 in CS13 of human embryos, which was a critical period of heart development. Our results showed that FGF8 (Fig. 7d) and FGF10 (Fig. 7e) were expressed in the OFT, further supporting the role of FGF8 and FGF10 in development of the OFT.

**Fig. 8:**
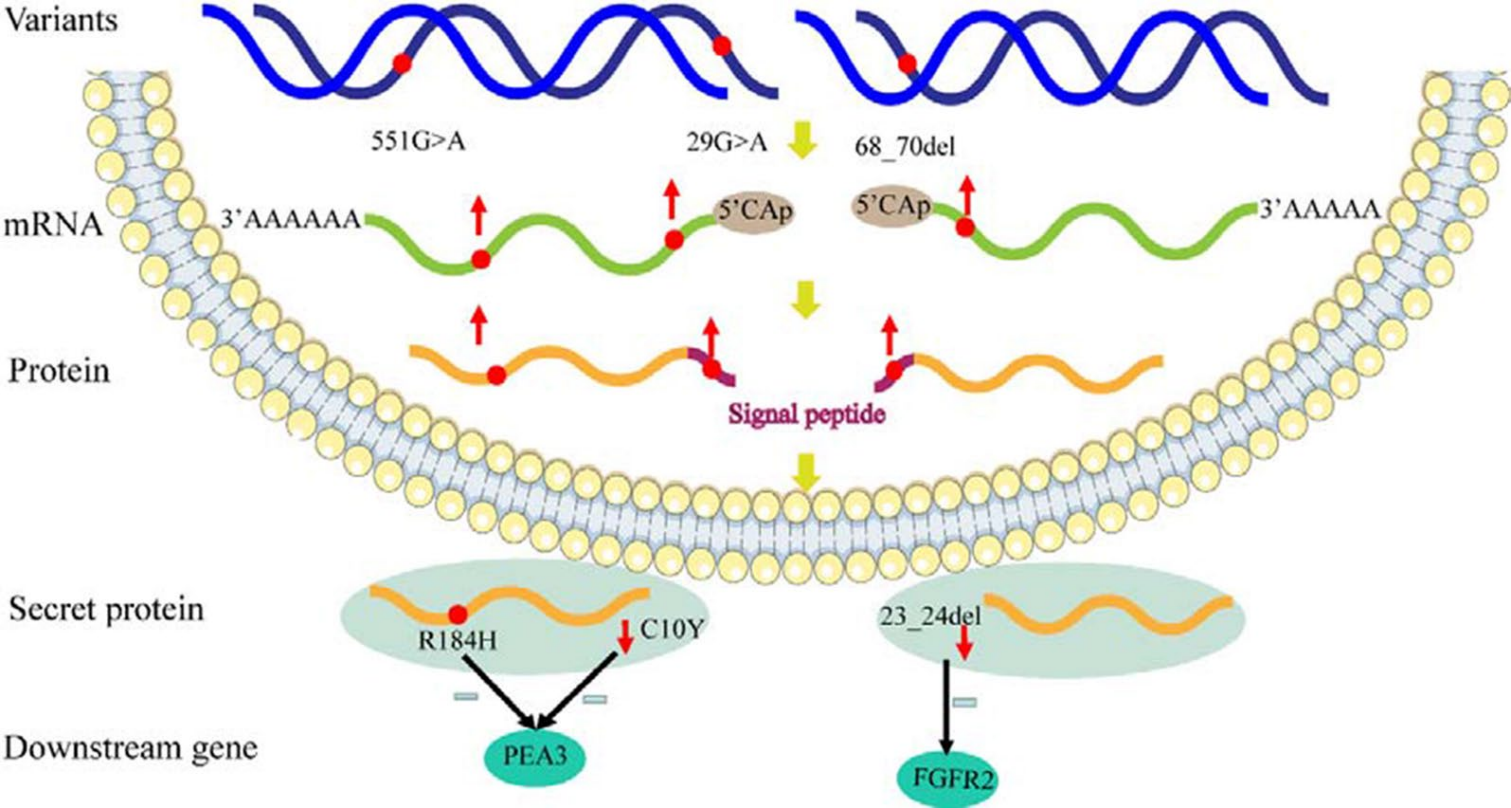
Scheme of the regulatory mechanisms involving FGF8 and FGF10 in the pathogenesis of CTDs. The upward arrow indicates increased expression, the downward arrow indicates decreased secretion, and the minus sign indicates inhibition

A schematic model of the functional impact of the identified mutations is shown in Fig. 8.

## Discussion

In our study of 585 patients with CHD, we identified two rare heterozygous mutations of FGF8, namely p.C10Y and p.R184H, in two unrelated patients both affected with TOF, and one deletion of FGF10, namely p.23_24del, in a patient with single atrium, single ventricle, complete atrioventricular canal defect, pulmonary stenosis, and pulmonary hypertension. These variants were predicted to be “possibly damaging” by bioinformatic analysis. Based on multiple sequence alignments, we found that these variations were highly evolutionary conserved, indicating that all these variations might have vital biological functions. Moreover, functional analysis revealed changes in the expression and function of these variant proteins compared with wild type. Both FGF8 and FGF10 increased the proliferation of HCM, and increased the expression of PEA3 and FGFR2, respectively [12, 26]. However, the mutations reduced both the HCM proliferation and the expression of PEA3 and FGFR2. Immunofluorescence staining demonstrated that none of these mutations affected the intracellular localization. However, ELISA analysis showed that both p.C10Y and p.23_24del mutations affected the secretion of FGF8 and FGF10. This effect could be explained by the localization of these mutations in the signal peptide regions. All of these indicated that although variants increased the expression of FGF8 and FGF10 in cell, their functions were reduced. Variants in the signal peptide regions (p.C10Y of FGF8 and p.23_24del of FGF10) might affect their function by reducing their secretion, whereas the p.R184H mutation of FGF8 might negatively affect the proliferation of HCM by reducing the expression of PEA3, eventually leading to CTD.

The vital implication of FGF8 and FGF10 in congenital cardiovascular malformations was identified in animal models. In mouse, FGF8 was shown to be required in the early stages of development, because FGF8 homozygous knockout embryos stopped growing during the gastrulation, and mesoderm migration was also impaired [27]. In addition, several studies have implicated FGF8 having vital roles during the cardiovascular development in chick and zebrafish [28, 29]. As is known, FGF10 is mainly expressed in cardiomyocytes [30]. Moreover, inactivation of the FGF10 signaling pathway has been reported to lead to a smaller, thin-walled heart [31]. In mice lacking FGF10, the position of the ventricular apex in the chest was shown to be incorrect, with a lack of pulmonary circulation [19]. The phenotype of mice with a double knockout of FGF8 and FGF10 has indicated a functional overlap of these proteins in the SHF mesoderm during development of the OFT and RV [20, 32, 33]. Consistent with this, we found that FGF8 was highly expressed in early differentiation of hESCs to cardiomyocytes, and especially during specification of the mesoderm stage. Whereas, the expression of FGF10 increased significantly on the fourth day of differentiation, peaking on the fifth day, and then decreased. Furthermore, both FGF8 and FGF10 were expressed in the OFT of human embryos. These findings further supported the idea that FGF8 and FGF10 played important roles in development of the OFT, consistent with results obtained from in animal models.

Our study had some limitations. For instance, all functional assays were performed in vitro. Besides, the differentiation of hESCs to cardiomyocytes does not completely simulate this phase of human embryonic development. Furthermore, the effects of these mutations on other biophysical and structural properties still require further research.

## Conclusion

In conclusion, we characterized three deleterious variants of FGF8 and FGF10 which were associated with the development of CTD, expanding the FGF mutation profile, and further supporting the pathogenic correlation between FGF mutations and CTD. Our findings might offer a basic understanding and open new areas toward elucidating the role of genes involved in the pathogenesis of CTD.

## Supplementary information

**Additional file 1: Table S1.** Primer pairs used to amplify the coding regions contain candidate variants.** Table S2.** Primer pairs used to screen of downstream target genes of FGF8 and FGF10.** Figure S1.** Cardiac ultrasound results in patients. A and B Echocardiography of a patient with TOF labeled F150, C Echocardiography of a patient with single atrium and single ventricle labeled S033.** Figure S2.** Screening of downstream target genes of FGF8 and FGF10. A and C Screening of downstream target genes of FGF8 in human cardiomyocytes and HEK293T cells, B and D Screening of downstream target genes of FGF10 in human cardiomyocytes and HEK293T cells (n = 3). GAPDH was used as an internal control.

## Data Availability

The data set analyzed in this study can be obtained from the corresponding author according to reasonable requirements.

## Abbreviations

CHDs: congenital heart diseases
CNC: cardiac neural crest
CS: carnegie stage
CTDs: conotruncal defects
DORV: double outlet of right ventricle
FGF: fibroblast growth factor
hESCs: human embryonic stem cells
IAA: interrupted aortic arch
OFT: outflow tract
PAA: pharyngeal arch arteries
PA/VSD: pulmonary atresia with ventricular septal defect
PTA: persistent truncus arteriosus
SHF: secondary heart field
TGA: transposition of the great arteries
TOF: Tetralogy of Fallot

## References

[1] O’Malley CD, Shaw GM, Wasserman CR, Lammer EJ (1996). Epidemiologic characteristics of conotruncal heart defects in California, 1987-1988. Teratology.

[2] Shah GS, Singh MK, Pandey TR, Kalakheti BK, Bhandari GP (2008). Incidence of congenital heart disease in tertiary care hospital. Kathmandu Univ Med J (KUMJ).

[3] Bouma BJ, Mulder BJ (2017). Changing landscape of congenital heart disease. Circ Res.

[4] Webb S, Qayyum SR, Anderson RH, Lamers WH, Richardson MK (2003). Septation and separation within the outflow tract of the developing heart. J Anat.

[5] Kodo K, Shibata S, Miyagawa-Tomita S, Ong SG, Takahashi H, Kume T, Okano H, Matsuoka R, Yamagishi H (2017). Regulation of Sema3c and the Interaction between Cardiac Neural Crest and Second Heart Field during Outflow Tract Development. Sci Rep.

[6] Jenkins KJ, Correa A, Feinstein JA, Botto L, Britt AE, Daniels SR, Elixson M, Warnes CA, Webb CL (2007). Noninherited risk factors and congenital cardiovascular defects: current knowledge: a scientific statement from the American Heart Association Council on Cardiovascular Disease in the Young: endorsed by the American Academy of Pediatrics. Circulation.

[7] Cooper WO, Hernandez-Diaz S, Arbogast PG, Dudley JA, Dyer S, Gideon PS, Hall K, Ray WA (2006). Major congenital malformations after first-trimester exposure to ACE inhibitors. N Engl J Med.

[8] Ornitz DM (2005). FGF signaling in the developing endochondral skeleton. Cytokine Growth Factor Rev.

[9] Kuro-o M (2008). Endocrine FGFs and Klothos: emerging concepts. Trends Endocrinol Metab.

[10] Beenken A, Mohammadi M (2009). The FGF family: biology, pathophysiology and therapy. Nat Rev Drug Discov.

[11] Itoh N, Ohta H, Nakayama Y, Konishi M (2016). Roles of FGF signals in heart development, health, and disease. Front Cell Dev Biol.

[12] Ornitz DM, Itoh N (2015). The Fibroblast Growth Factor signaling pathway. Wiley Interdiscip Rev Dev Biol.

[13] Park EJ, Ogden LA, Talbot A, Evans S, Cai CL, Black BL, Frank DU, Moon AM (2006). Required, tissue-specific roles for Fgf8 in outflow tract formation and remodeling. Development.

[14] Ilagan R, Abu-Issa R, Brown D, Yang YP, Jiao K, Schwartz RJ, Klingensmith J, Meyers EN (2006). Fgf8 is required for anterior heart field development. Development.

[15] Hu T, Yamagishi H, Maeda J, McAnally J, Yamagishi C, Srivastava D (2004). Tbx1 regulates fibroblast growth factors in the anterior heart field through a reinforcing autoregulatory loop involving forkhead transcription factors. Development.

[16] Abu-Issa R, Smyth G, Smoak I, Yamamura K, Meyers EN (2002). Fgf8 is required for pharyngeal arch and cardiovascular development in the mouse. Development.

[17] Frank DU, Fotheringham LK, Brewer JA, Muglia LJ, Tristani-Firouzi M, Capecchi MR, Moon AM (2002). An Fgf8 mouse mutant phenocopies human 22q11 deletion syndrome. Development.

[18] Kelly RG, Brown NA, Buckingham ME (2001). The arterial pole of the mouse heart forms from Fgf10-expressing cells in pharyngeal mesoderm. Dev Cell.

[19] Marguerie A, Bajolle F, Zaffran S, Brown NA, Dickson C, Buckingham ME, Kelly RG (2006). Congenital heart defects in Fgfr2-IIIb and Fgf10 mutant mice. Cardiovasc Res.

[20] Watanabe Y, Miyagawa-Tomita S, Vincent SD, Kelly RG, Moon AM, Buckingham ME (2010). Role of mesodermal FGF8 and FGF10 overlaps in the development of the arterial pole of the heart and pharyngeal arch arteries. Circ Res.

[21] Vitelli F, Taddei I, Morishima M, Meyers EN, Lindsay EA, Baldini A (2002). A genetic link between Tbx1 and fibroblast growth factor signaling. Development.

[22] Zhang X, Xu Y, Liu D, Geng J, Chen S, Jiang Z, Fu Q, Sun K (2015). A modified multiplex ligation-dependent probe amplification method for the detection of 22q11.2 copy number variations in patients with congenital heart disease. BMC Genomics.

[23] Khalil AA, Sivakumar S, Lucas FAS, McDowell T, Lang W, Tabata K, Fujimoto J, Yatabe Y, Spira A, Scheet P, Nemer G, Kadara H (2017). TBX2 subfamily suppression in lung cancer pathogenesis: a high-potential marker for early detection. Oncotarget.

[24] Xie H, Hong N, Zhang E, Li F, Sun K, Yu Y (2019). Identification of rare copy number variants associated with pulmonary atresia with ventricular septal defect. Front Genet.

[25] Devillers-Thiery A, Kindt T, Scheele G, Blobel G (1975). Homology in amino-terminal sequence of precursors to pancreatic secretory proteins. Proc Natl Acad Sci USA.

[26] Roehl H, Nusslein-Volhard C (2001). Zebrafish pea3 and erm are general targets of FGF8 signaling. Curr Biol.

[27] Sun X, Meyers EN, Lewandoski M, Martin GR (1999). Targeted disruption of Fgf8 causes failure of cell migration in the gastrulating mouse embryo. Genes Dev.

[28] Alsan BH, Schultheiss TM (2002). Regulation of avian cardiogenesis by Fgf8 signaling. Development.

[29] Reifers F, Walsh EC, Leger S, Stainier DY, Brand M (2000). Induction and differentiation of the zebrafish heart requires fibroblast growth factor 8 (fgf8/acerebellar). Development.

[30] Rochais F, Sturny R, Chao CM, Mesbah K, Bennett M, Mohun TJ, Bellusci S, Kelly RG (2014). FGF10 promotes regional foetal cardiomyocyte proliferation and adult cardiomyocyte cell-cycle re-entry. Cardiovasc Res.

[31] Vega-Hernandez M, Kovacs A, De Langhe S, Ornitz DM (2011). FGF10/FGFR2b signaling is essential for cardiac fibroblast development and growth of the myocardium. Development.

[32] Zelarayan LC, Vendrell V, Alvarez Y, Dominguez-Frutos E, Theil T, Alonso MT, Maconochie M, Schimmang T (2007). Differential requirements for FGF3, FGF8 and FGF10 during inner ear development. Dev Biol.

[33] Ohuchi H, Nakagawa T, Itoh N, Noji S (1999). FGF10 can induce Fgf8 expression concomitantly with En1 and R-fng expression in chick limb ectoderm, independent of its dorsoventral specification. Dev Growth Differ.

