# Supplementary material for: Mutations in fibroblast growth factor (FGF8) and FGF10 identified in patients with conotruncal defects
Source: J Transl Med. 2020 Jul 14;18:283. doi: 10.1186/s12967-020-02445-2 (PMC7362408; doi:10.1186/s12967-020-02445-2)

Table S1. Primer pairs used to amplify the coding regions contain candidate variants

| Gene | Candidate variants | Primer orientation | Primer sequences | Product size (bp) |
| --- | --- | --- | --- | --- |
| FGF8 | 29G>A | F | TGAGTTGCCTGCTGTTGC | 663 |
|  |  | R | TGAAGGGCGGGTAGTTGAG |  |
|  | 551G>A | F | GGGAGCCCCAGGGTGTCT | 423 |
|  |  | R | TGTGGGTGAAGGCCATGT |  |
| FGF10 | 68_70del | F | CGGCTGCTGCTGCTGCTTTT | 560 |
|  |  | R | CCATTGGAAGAAAGTGAGCAGAG |  |

Table S2. Primer pairs used to screen of downstream target genes of FGF8 and FGF10

| Gene | Primer orientation | Primer sequences |
| --- | --- | --- |
| BMP15 | F | TGTGAACTCGTGCTTTTCATGG |
|  | R | CTCAATCAGGGGCAAAGTAGG |
| ER81 | F | CTGAACCCTGTAACTCCTTTCC |
|  | R | AGACATCTGGCGTTGGTACATA |
| FGF22 | F | GGGAGCGCATCGAAGAGAAC |
|  | R | CTGTGAGGCGTAGGTGTTGTG |
| FGFR2 | F | AGCACCATACTGGACCAACAC |
|  | R | GGCAGCGAAACTTGACAGTG |
| HDAC | F | CTACTACGACGGGGATGTTGG |
|  | R | GAGTCATGCGGATTCGGTGAG |
| NOTCH1 | F | GAGGCGTGGCAGACTATGC |
|  | R | CTTGTACTCCGTCAGCGTGA |
| PEA3 | F | GATGAAAGCCGGATACTTGGAC |
|  | R | TTCGCGCAAGCTCCCATTT |
| SOX2 | F | GCCGAGTGGAAACTTTTGTCG |
|  | R | GGCAGCGTGTACTTATCCTTCT |
| SOX9 | F | AGCGAACGCACATCAAGAC |
|  | R | CTGTAGGCGATCTGTTGGGG |
| SOX10 | F | CCTCACAGATCGCCTACACC |
|  | R | CATATAGGAGAAGGCCGAGTAGA |
| SNAIL | F | TCGGAAGCCTAACTACAGCGA |
|  | R | AGATGAGCATTGGCAGCGAG |
| TBX1 | F | ACGACAACGGCCACATTATTC |
|  | R | CCTCGGCATATTTCTCGCTATCT |
| TBX2 | F | CCCCTTCAAGGTGCGAGTC |
|  | R | TCAGCGGCTACAATGTCCATC |
| WNT3A | F | AGCTACCCGATCTGGTGGTC |
|  | R | CAAACTCGATGTCCTCGCTAC |
| WNT4 | F | AGGAGGAGACGTGCGAGAAA |
|  | R | CGAGTCCATGACTTCCAGGT |

Figure S1 Cardiac ultrasound results in patients. A and B Echocardiography of a patient with TOF labeled F150, C Echocardiography of a patient with single atrium and single ventricle labeled S033.


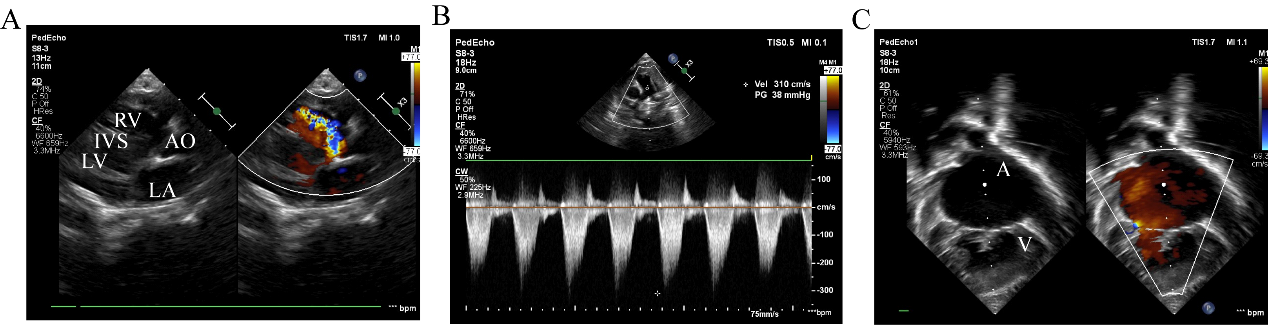


Figure S2 Screening of downstream target genes of FGF8 and FGF10. A and C Screening of downstream target genes of FGF8 in human cardiomyocytes and HEK293T cells, B and D Screening of downstream target genes of FGF10 in human cardiomyocytes and HEK293T cells (n = 3). GAPDH was used as an internal control.


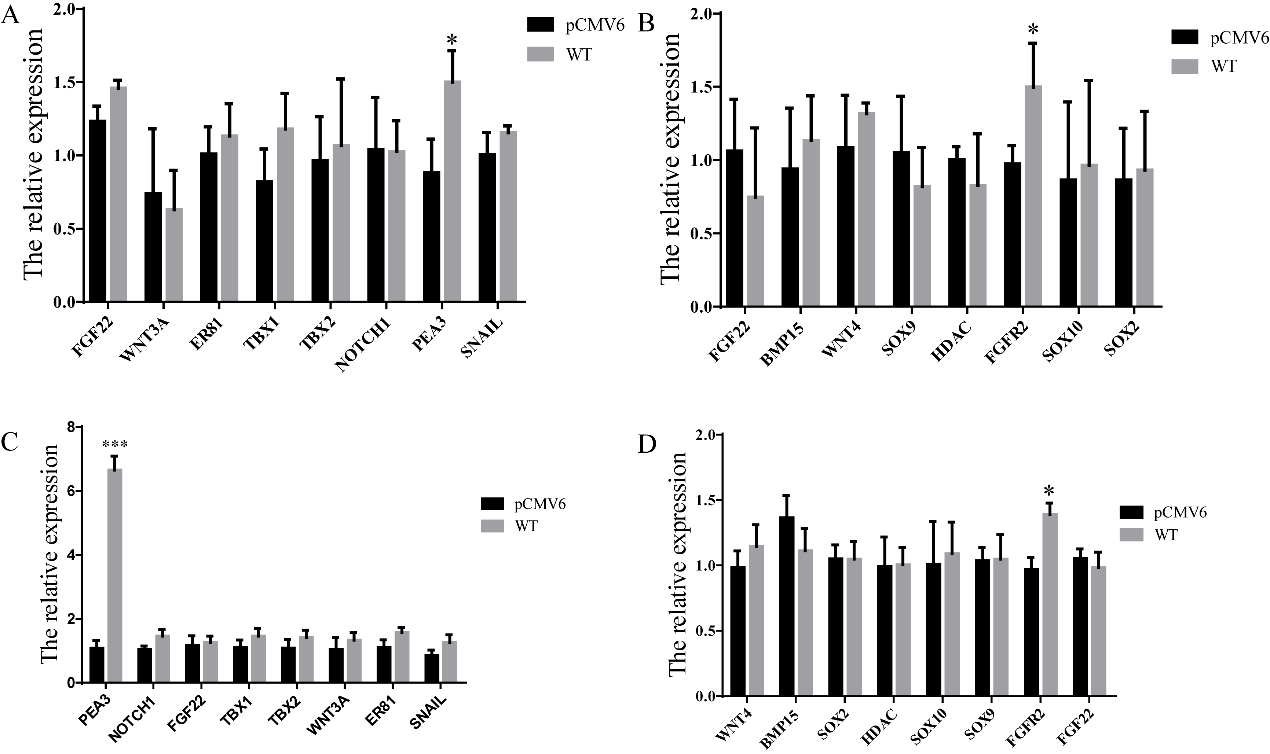

Supplement: Supplementary file 1 — Additional file 1: Table S1. Primer pairs used to amplify the coding regions contain candidate variants. Table S2. Primer pairs used to screen of downstream target genes of FGF8 and FGF10. Figure S1. Cardiac ultrasound results in patients. A and B Echocardiography of a patient with TOF labeled F150, C Echocardiography of a patient with single atrium and single ventricle labeled S033. Figure S2. Screening of downstream target genes of FGF8 and FGF10. A and C Screening of downstream target genes of FGF8 in human cardiomyocytes and HEK293T cells, B and D Screening of downstream target genes of FGF10 in human cardiomyocytes and HEK293T cells (n = 3). GAPDH was used as an internal control. [file 12967_2020_2445_MOESM1_ESM.docx]
